# Replicability and replication in the humanities

**DOI:** 10.1186/s41073-018-0060-4

**Published:** 2019-01-09

**Authors:** Rik Peels

**Affiliations:** Philosophy Department, Faculty of Humanities, Vrije Universiteit Amsterdam, De Boelelaan 1105, 1081 HV Amsterdam, The Netherlands

**Keywords:** Humanities, Normativity, Replicability, Replication, Replication crisis, Uniqueness

## Abstract

A large number of scientists and several news platforms have, over the last few years, been speaking of a replication crisis in various academic disciplines, especially the biomedical and social sciences. This paper answers the novel question of whether we should also pursue replication in the humanities. First, I create more conceptual clarity by defining, in addition to the term “humanities,” various key terms in the debate on replication, such as “reproduction” and “replicability.” In doing so, I pay attention to what is supposed to be the object of replication: certain studies, particular inferences, of specific results. After that, I spell out three reasons for thinking that replication in the humanities is not possible and argue that they are unconvincing. Subsequently, I give a more detailed case for thinking that replication in the humanities is possible. Finally, I explain why such replication in the humanities is not only possible, but also desirable.

## Background

Scientists and various news platforms have, over the last few years, increasingly been speaking of a *replication crisis* in various academic disciplines, especially the biomedical 1 and social sciences.^2^ The main reason for this is that it turns out that large numbers of studies cannot be replicated, that is (roughly), they yield results that appear not to support, or to count against, the validity of the original finding.^3^ This has been and still is an important impulse for composing and adapting various codes of research integrity. Moreover, in December 2017, the National American Academies convened the first meeting of a new study committee that will, for a period of 18 months, study “Reproducibility and Replicability in Science,” a project funded by the National Science Foundation.^4^ Finally, over the last few years, various official reports on replication have been published. At least five of them come to mind:

- The 2015 report by the National Science Foundation: *Social, Behavioral, and Economic Sciences Perspectives on Robust and Reliable Science*
- The 2015 symposium report by the Academy of Medical Sciences: *Reproducibility and Reliability of Biomedical Research*
- The 2016 workshop report by the National Academies of Sciences: *Statistical Challenges in Assessing and Fostering the Reproducibility of Scientific Results* [1]
- The 2016 report by the Interacademy Partnership for Health: *A Call for Action to Improve the Reproducibility of Biomedical Research*
- The 2018 advisory report by the Royal Netherlands Academy of Arts and Sciences *Replication Studies*^5^

These documents state what the problem regarding replication is, they explain how we should think of the nature and value of replication, and they make various recommendations as to how to improve upon replicability. There are many causes for lack of replicability and failure to successfully replicate upon attempting to do so. Among them are (i) fraud, falsification, and plagiarism, (ii) questionable research practices, partly due to unhealthy research systems with perverse publication incentives, (iii) human error, (iv) changes in conditions and circumstances, (v) lack of effective peer review, and (vi) lack of rigor.^6^ Thus, we also need a wide variety of measures to improve on replicability. In this article, I will take each of the reports mentioned above into consideration, but pay special attention to the KNAW report, since it is the most recent one and it has taken the findings of the other reports into account.

The issue of replicability and replication in academic research is important for various reasons. Let me mention four of them: (i) results that are consistently replicated are likely to be true, all else being equal, that is, controlling for such phenomena as publication bias and assuming that the other assumptions in the relevant theory or model are valid, (ii) replicability prevents the waste of (financial, time, etc.) resources, since studies that cannot be consistently replicated are less likely to be true, (iii) results that are not replicable are, if they are applied, more likely to cause harm to individuals, animals, and society (e.g., by leading to mistaken economic measures or medicine that is detrimental to people’s health), and (iv) if too many results turn out not to be replicable, upon attempting to replicate them, that will gradually erode public trust in science.^7^

Now, reports about replication focus on various *quantitative empirical sciences*.^8^ The KNAW Advisory Report, for instance, makes explicit that it is confined to the medical sciences, life sciences, and psychology.^9^ These reports, though invite researchers from other disciplines to consider the relevance of these documents and recommendations for their own fields. That is precisely the purpose of this paper: to explore to what extent replication is possible and desirable in another important field of scholarly activity, namely the *humanities*. After all, many humanistic disciplines, such as history, archeology, linguistics, and art theory are thoroughly *empirical*: they are based on the collection of data (as opposed to the deductive lines of reasoning that we find in mathematics, logic, parts of ethics, and metaphysics). This naturally leads to the question whether replication is also possible in the humanities.

How we should think of replication in the humanities is something that has not received any attention so far, except for a couple of articles that I co-authored with Lex Bouter.^10^ Maybe this is because it is questionable whether replication is even *possible* in the humanities. There are various reasons for this. First, the study objects in the humanities are often unique phenomena, such as historical events, so that it is not clear in what sense one could replicate a study. Second, one might think that various methods in the humanities, such as the hermeneutical method in studying a text, do not lend themselves well to replication—at least not as well as certain methods in the quantitative empirical sciences, where one can carry out an experiment with similar data under similar circumstances. Third, the objects of humanistic research, in opposition to the objects of research in the natural sciences, are often object with *meaning* and *value*, objects such as paintings, texts, statues, and buildings—in opposition to, say, such objects as atoms and viruses that are studied in the natural sciences. One might think that the inevitably normative nature of these humanistic objects makes replication impossible. It remains to be seen, though, whether these objections hold water. I return to each of them below.

## Introduction

In order to answer the question of whether replication is possible and, if so, desirable in the humanities, I first create more conceptual clarity by defining, in addition to the term “humanities,” various key terms in the debate on replication, such as “reproduction” and “replicability.” In doing so, I pay attention to what is supposed to be the object of replication: certain studies, particular inferences, of specific results. After that, I lay out three reasons for thinking that replication in the humanities is not possible and argue that they are unconvincing. Subsequently, I give a more detailed case for thinking that replication in the humanities is possible. Finally, I explain why such replication in the humanities is not only *possible*, but also *desirable*.

## Defining the key terms

We can be rather brief about the term “humanities.” There is a debate on what should count as a humanistic discipline and what not. Rather than entering that debate here, I will simply stipulate that, for the sake of argument, I take the following disciplines to belong to the humanities: anthropology; archeology; classics; history; linguistics and languages; law and politics; literature; the study of the performing arts, such as music, theater, and dance; the study of the visual arts, such as drawing, painting, and film; philosophy; theology; and religious studies. This captures what most people take to fall under the umbrella of “humanities” and that will do for the purposes of this paper.^11^

Let us now move on to replication. There are at least two complicating factors when it comes to the issue of replication in the humanities: there is a wide variety of terms and many of these terms have no definition that is widely agreed upon. I have the following eight terms in mind: “replication study,” “replicability,” “replication,” “reproduction,” “reproducibility,” “robustness,” “reliability,” and “verifiability.” Here, I will put the final three terms, namely “robustness,” “reliability,” and “verifiability” aside, since the points I want to make about replication in the humanities do not depend on them.^12^ Also, I take “replication” and “reproduction” to be synonyms, as I do “replicability” and “reproducibility.”^13^ I will, therefore, focus on the three remaining terms, to wit “replication studies,” “replicability,” and “replication.”

Let us define “replication study” as follows:

- Replication study

**A replication study is a study that is an independent repetition of an earlier, published study, using sufficiently similar methods (along the appropriate dimensions) and conducted under sufficiently similar circumstances**.^14^

Clearly, this definition requires some explanation. First, it counts both studies that are meant as *close* or *exact* replication and studies designed as *conceptual* replication as replication studies. There are, of course, crucial differences between these kinds of replication, but they both count as replication studies and that is exactly what the above definition is meant to capture. A recent call for replication studies by the Netherlands Organization for Scientific Research (NWO), for instance, distinguishes three kinds of replication^15^:

1. Replication with *existing data* and the *same research protocol* and the same research question: repeated analysis of the datasets from the original study with the original research question (sometimes more narrowly referred to as a “reproduction”).
2. Replication with a *new data* collection and with the *same research protocol* and the same research question as the original study (often referred to as a “direct replication”).
3. Replication with *new data* and with a *new or revised research protocol*^16^: new data collection with a different design from the original study in which the research question remains unchanged compared to that of the original study (often referred to as a “conceptual replication”).^17^

An advantage of the above definition of “replication study” is that it captures these three varieties of replication studies. It is, of course, perfectly compatible with my definition to make these further distinctions among varieties of replication studies.

Second, the definition states that the new study should in some sense be *independent* from the original study. Unfortunately, reports on replication usually do not define what it is for a study to be independent from an earlier one.^18^ It seems to me that the right way to understand “independence” here is that the new study should not in any way depend *on the results of the original study*.

However, can we be more precise about how the results of the new study should not *depend* on those of the original study? The most obvious meaning of this phrase is that the new study should not take all the original results *for granted*—that is, it should not assume their truth or correctness in its line of reasoning (even though, it can of course do so merely *for the sake of argument*). Dependence, however, is a matter of degree: one can, for instance, assume *certain results* or *certain aspects of certain results* in order to replicate other results or other aspects of results. Below, we return to the issue of degrees when we consider in what sense results of the new study should *agree* with the results of the original study.

This means that various other kinds of dependence are perfectly legitimate for a replication study. For example, the new study can depend on the same *instruments* as those used in the original study, on the same *research protocol* (e.g., in a repetition of an earlier study), and, in some cases, even on the *original researchers* or at least partly so in the case of a collaborative team with the original researchers and new researchers. It can perfectly well *depend on* these things in that it is no problem if the original study and the new study have the same instruments, the same research protocol, and consists of the same group of researchers—at least for *some* kinds of replication.

Third and finally, the definition states that the methods used and the circumstances in which the study is carried out should be “sufficiently similar.” That means that they need not be identical—that may be the case (or something very close to that), but that is not required for a replication study. It also means that they should not be completely different—that is excluded by its being a replication study. But exactly when are they “sufficiently similar?”

This is a complex issue that others have addressed in detail. For instance, Etienne LeBel and others provide a replication taxonomy that understands replication as a graded phenomenon: it ranges from an exact replication (all facets that are under the researchers’ control are the same) to a very far replication (independent variables (IV) or dependent variables (DV) constructs are different), with, respectively, very close replication, close replication, and far replication in-between. The design facets that their taxonomy pays attention to are such things as effect or hypothesis, IV construct, DV construct, operationalization, population (e.g., seize), IV stimuli, DV stimuli, procedural details, such as task instructions and font size, physical setting, and contextual variables (they indicate that the list can be extended).^19^ What this goes to show is that replication is a matter of degree and that in assessing the epistemic status of a replication, one should try to locate it on a replication continuum.

This brings us to the second key term, “replicability.” It seems to me that this term is used in two crucially different ways, in the KNAW Advisory Report as well as in the broader literature on replication studies. In order to keep things clear, I would like to distinguish the two and will refer to the former as “replicability” and to the latter as “replication.” I define them as follows:

- Replicability

**A study is having certain features such that a replication study of it could be carried out**.

- Replication

**A study is being such that a repetition of it has successfully been carried out, producing results that agree with the original study**.^20^

Some philosophers of science and scholars in research integrity use the term “transparency” for what I dub “replicability” here.^21^ Clearly, replicability, as I understand it here, has much to do with transparency: a study can be replicated only if the researchers are sufficiently transparent about the data, the method, the inferences, and so on. Still, I prefer to use the term “replicability” rather than “transparency,” given the purposes of this paper. This is because some humanistic scholars, as we shall see below, think that studies can be perfectly transparent and yet such that they cannot be replicated. If so, they are not replicable, but not because of any scholarly shortcoming. Rather, it would be the nature of the beast (a humanistic study, or a particular kind of humanistic study, such as one about value or meaning) that prevents the possibility of replication.

Thus, replicability is a desideratum for at least many studies in the quantitative empirical sciences (I return to the humanities below): we want them to be set-up and described in such a way that, in principle, we could carry out a replication study. Precise definitions, a clear description of the methodology (in the research protocol), a clear overview of the raw data, a lucid analysis of the data, and so on, all contribute to the replicability of a study. One of the things the replication crisis has made clear is that many studies in the empirical sciences fail to meet the criterion of replicability: we cannot carry out a replication study of them, since the key terms are not even sufficiently clearly defined, the method is underdescribed, the discussion is not transparent, the raw data are not presented in a lucid way, or the analysis of the data is not clearly described.

*Replicability* should be clearly distinguished from *replication*. Replication entails replicability (you cannot replicate what is not replicable), but requires significantly more, namely that a successful replication has actually taken place, producing results that agree with the results of the original study. Thus, in a way this distinction is similar to Karl Popper’s famous distinction between falsifiability and falsification.^22^ Falsifiability is a desideratum for any scientific theory: very roughly, a theory should be such that it is *in principle* falsifiable. Falsification entails falsifiability, but goes a step further, because a falsified theory is a theory that is not only falsifiable, but that has in fact also been falsified. I said “roughly,” because, as Brian Earp has argued in more detail, things are never so simple when it comes to falsification: even if an attempt at falsification has taken place and the new data seem to count against the original hypothesis, one might often just as well, say, question an auxiliary assumption, consider whether a mistake was made in the original study, or wonder whether perhaps the original effect *is* a genuine effect but one that can only be obtained under specific conditions.^23^ Nevertheless, falsification is often still considered as a useful heuristic in judging the strength of a hypothesis.^24^ Now, the obvious difference with the issue at hand is that, even though both falsifiability and replicability are desiderata, replication is a good thing, because it makes it, all else being equal, likely that results are true, whereas falsification is in a sense a bad thing, because it makes it likely that a theory is false.^25^

A replication study, then, is a study that aims at replication. Such replication may fail either because the original study turns out not to be replicable in the first place or because, even though it is replicable, a successful replication does not occur. A successful replication occurs if the results of the new study *agree* with those of the original study or, slightly more precisely, if the results of the two studies are commensurate. Exactly what is it, though, for results to be commensurate? As several reports on replication point out^26^ it is not required that the results are identical—that would be too demanding in, say, many biomedical sciences. Again, it seems that “agreeing” is a property of results that *comes in degrees*. More precisely, we can distinguish at least the following senses, in order of increasing strength:

- The studies’ conclusions have the same *direction* (e.g., both studies show a positive correlation between X and Y);
- The studies’ conclusions have the same *direction* and the studies have a *similar effect size* (e.g., in both studies, Y is three times as large with X as it is with non-X; in some disciplines: the relative risk is three (RR = 3));
- The studies’ conclusions have the same *direction*, and the studies have a *similar effect size* and a *similar p value*, *confidence interval*, or *Bayes factor* (e.g., for both studies, RR = 3 (1.5–5.0)).^27^

The stronger the criterion for the sense in which studies results “agree,” the lower—ceteris paribus—the percentage of successful replications will be, at least when it comes to quantitative empirical research.

Now, what does a typical replication study look like? The aforementioned KNAW Advisory Report sketches four characteristics: it “(a) is carried out by a team of independent investigators; (b) generates new data; (c) follows the original protocol closely and justifies any deviations; and (d) attempts to explain the resulting degree of reproducibility.”.^28^ Thus, even though, as I pointed out above, independence does not require that the replication study be carried out by different researchers than the original study, this is nonetheless often the case. Below, we will explore to what extent we encounter the combination of these characteristics in the humanities.

Before we move on to replicability and replication in the humanities, I would like to make two preliminary points. First, we should note that it follows from the definitions of “replicability” and “replication” given in this section that both replicability and replication are a matter of *degree*.^29^ Replication studies can be pretty much identical to the original study, but very often there are slight or even somewhat larger alterations in samples, instruments, conditions, researcher skills, the body of researchers, and sometimes even changes in the method. One can change the method, for instance, in order to explore whether a similar finding can be obtained by way of a rather different method, or a finding that would similarly support one of the relevant underlying hypotheses, at least if the auxiliary assumptions are also met. Every replication study can be located on a continuum that goes from being a replication almost identical to the original study to hardly being a replication at all. The closer the replication study topic is to the topic of the original study, the more it counts as a replication study, and, similarly, for method, samples, conditions, and so on. How we ought to balance these various factors in assessing how much of a replication a particular study is, is a complicated matter that we need not settle here; all we need to realize is that replication is something that comes in degrees. As I briefly spelled out above, in laying out Etienne LeBel’s replication taxonomy, a study can be more or less of a replication of an original study.^30^

Second, exactly what is it that should be replicable in a good replication study? There are at least three candidates here: the study as a whole, the inferences involved in the study, and the results of the study.^31^ I will focus on the replicability of a study’s *results*. After all, as suggested in our discussion above, we want to leave room for the possibility of a direct replication (which uses new data, so that the study as a whole is not replicated), and a conceptual replication (which uses new data and a new research protocol, so that neither the study as a whole nor its specific inferences are replicated). This means that a study is replicable if a new study can be carried out, producing results that might agree with those of the original study in the sense specified above.

## Potential obstacles to replication in the humanities

Now, one might think that, in opposition to the quantitative empirical sciences, such as the biomedical sciences, the humanities are not really suited for the phenomenon of replication. In this section, I discuss three arguments in support of this claim.

1. The first objection to the idea that replication is possible in the humanities is that, frequently, the study object in the humanities is *unique*^32^: there was one French Revolution in 1789–1799, there is one novel of Virginia Woolf named *To the Lighthouse* (1928), pieces of architecture, such as Magdalen College’s library in Oxford, are unique, and so on. Viruses, atoms, leg fractures, Borneo’s rhinos, economic measures, and many other study objects in the empirical sciences, have multiple instances. In a replication study one can investigate a different instance or token than the one studied in the original study; an instance or token of the same type.

However, this objection fails for two reasons. On the one hand, many study objects in the humanities *do* have multiple instances. On the other hand, quite a few study objects in the empirical sciences are unique. As to the former: Virginia Woolf’s *To the Lighthouse* is unique, but it is also one of many instances of novels using a stream-of-consciousness-narrative technique; the French Revolution is unique, but it is an instance of a social revolution, of which the American Revolution in 1775–1783 and the Russian Revolution in 1917 are other examples. Magdalen College library can be compared to other college libraries in Oxford, to other libraries across the country, and to other buildings in the late fifteenth century. And so on. Parts of linguistics study grammatical structures that, by definition, have many instances, as will be clear from any introduction to morphosyntax.^33^ As to the quantitative empirical sciences: the big bang, the coming into existence of life on earth, space-time itself, and many other phenomena studied in the empirical sciences are unique phenomena: there is only one instance of them. Thus, the idea that the empirical sciences study phenomena that have multiple instances, whereas the humanities study unique phenomena is, as a general claim, untenable.

Second and more importantly, whether or not the object of study is unique or not is *immaterial* to the issue of the replicability of a study on that object. After all, one may study an object several times and studying it several times may even generate new data (a typical property of many replication studies, as we noted in the previous section). For example, even though the French revolution was a unique historical event (or a unique series of events), that event comprises so many data, laid down in artifacts, literary accounts, paintings, and so on, that it is possible to repeat a particular method—say, studying a text—and even discover new things about that unique event.^34^

2. A second argument against the idea that replication is possible in the humanities is that many methodologies that are employed in the humanities do not lend themselves well to replication. By replicating an *empirical* study, say, on whether or not patients with incident migraine, in comparison with the general population, have higher absolute risks of suffering from myocardial infarction, stroke, peripheral artery disease, atrial fibrillation, and heart failure^35^ one can, in principle, apply the same method or a similar method to *new patients* (say, a population from a different country). One can generate *new data*, thus making it likely—if replications consistently deliver sufficiently similar results—that the original results are true. One might think that no such thing takes place when one employs the methods of the humanities.

In response to this objection, I think it is important to note that there is a *wide variety of methods* used in the humanities. Among them are: more or less formal logic (in philosophy, theology, and law), literary analysis (in literary studies, philosophy, and theology), historical analysis (in historical studies, philosophy, and theology) and various narrative approaches^36^ (in historical studies), constructivism (in art theory, for instance), Socratic questioning (in philosophy), methods involving empathy (in literary studies and art studies), conceptual analysis (in philosophy and theology), the hermeneutical method (in any humanistic discipline that involves careful reading of texts, such as law, history, and theology), interviews (e.g., in anthropology), and phenomenology (in philosophy). This is important to note, because, as I pointed out above, I only want to argue that replication is possible in the humanities *to the extent that they are empirical*. Replication may not be possible in disciplines that primarily use a *deductive* method and that do not collect and analyze data, such as logic, mathematics, certain parts of ethics, and metaphysics. This leaves plenty of room for replication in disciplines that are empirical, such as literary studies, linguistics, history, and the study of the arts.

Take the hermeneutical method. Does reading a text again make it, all else being equal, likely that one’s interpretation is correct? It seems to me the answer here has to be positive. There are at least two reasons for that. First, one may have made certain mistakes in one’s original reading and interpretation: faulty reading, sloppy analysis, forgetting relevant passages, and so on, on the first occasion may play a role. If one’s second interpretation differs from the first, one will normally realize that and revisit the relevant passage, comparing which of the two interpretations is more plausible. This will generally increase the likelihood that one comes to a correct interpretation of, say, the relevant passage in Ovid. Second, if one re-reads certain passages that will be with new background beliefs, given that humanistic scholars gradually acquire more knowledge in the course of their lives. That may lead to a new interpretation. Unless one thinks that new beliefs are as likely to be false as true—which seems implausible—carefully re-reading a passage with relevant new background beliefs and coming to the same result increases the likelihood of truth of one’s interpretation. These two points apply *a forteriori* when *other* rather than *the same* humanistic scholars apply the same method of interpretation (the hermeneutical approach or a historical-critical methodology) to the same text. They will come to an interpretation and compare it with the original one; if it differs, they are likely to revisit relevant passages and, thereby, filter out forgetting, sloppiness, and mistakes.^37^ And, of course, they bring new background knowledge to a text. That as well makes it likely that when a study is consistently replicated, then, all else being equal, the original study results are likely to be true.

3. A third objection to the idea that replication is possible in the humanities, is that many of the study objects in the humanities are *normative* in the sense that they are objects of value and meaning, whereas this is not the case in many of the natural and biomedical sciences. René van Woudenberg, for instance, has argued in a recent paper that the objects of the humanities are such meaningful and/or valuable things as words, sentences, perlocutionary acts, buildings and paintings, music, and all sorts of artifacts. Molecules, laws of nature, diseases, and the like lack that specific sort of meaning and value.^38^

In reply, let me say that I will grant the assumption that the humanities are concerned with objects of value and meaning, whereas the sciences are not (or at least not with *those aspects* of those objects). I think this is not entirely true: some humanistic disciplines, such as metaphysics, are also concerned with objects that do not have meaning or value, such as numbers or the nature of space-time. It will still be true for most humanistic disciplines, though.

However, this point is not relevant for the issue of replication. This can be seen by considering, on the one hand, a scenario in which knowledge about value and meaning is *not* possible and, on the other, a scenario in which knowledge about value and meaning *is* possible. First, imagine that it is *impossible* to uncover knowledge about objects with value and meaning and specifically about those aspects of those objects that concern value and meaning. One may think, for instance, that there are no such facts about value and meaning^39^ or that they are all socially constructed, so that it would not be right to say that the humanities can *uncover* them.^40^ This is, of course, a controversial issue. Here, I will not delve into this complex issue, which would merit a paper or more of its own. Rather, I would like to point out that if it is indeed impossible to uncover knowledge about value and meaning, then that is a problem for the humanities *in general*, and not specifically for the issue of *replication* in the humanities. For, if there is no value and meaning, or if all value and meaning is socially constructed and the humanities can, therefore, not truly *uncover* value and meaning, one may rightly wonder to what extent humanistic scholarship as an academic discipline is still possible.

Now, imagine, on the other hand, that it *is* possible to uncover knowledge about objects with value and meaning and even about those aspects of those objects that specifically concern value and meaning. Then, it seems possible to uncover such knowledge and understanding about the aspects that involve value and meaning *multiple times* for the same or similar objects. And that would mean that in that case, it would very well be possible to carry out a replication study that involves conclusions about value and meaning. Of course, given the fact that the objects have value and meaning, it might sometimes be harder to reach agreement among scholars. After all, background assumptions bear heavily on issues concerning value and meaning. However, as several examples below show, agreement about issues concerning value and meaning is still quite often possible in the humanities.

I conclude that three main reasons for thinking that replication is not possible in the humanities do not hold water.

## A positive case for the possibility of replication in the humanities

So far, I have primarily deflected three objections to the possibility of replication in the humanities. Is there actually also a more detailed, positive case to be made for the possibility of replication in the humanities? Yes. In this section, I shall provide such a case.

My positive, more substantive case is an inductive one: there are many cases of replication studies in the humanities in the sense stipulated above: a study’s being such that a replication of it has successfully been carried out, producing results that agree with the original study. Moreover, they often meet the four stereotypical properties mentioned above: (a) they are carried out by a team of independent investigators; (b) they generate new data; (c) they follow the original protocol (or, at least, method description) closely and justify any deviations; and (d) attempt to explain the resulting degree of reproducibility.

Here is an example: re-interpreting Aurelius Augustine’s (354–430 AD) writings in order to see to what extent he continued to embrace or rejected Gnosticism. Using the hermeneutical method^41^—with such principles as that one should generally opt for interpretations of passages that make the text internally coherent, that one should, in interpreting a text, take its genre into account, and so on—and relevant historical background knowledge, it has time and again been confirmed that Augustine came to reject the basic tenets of Gnosticism—such as the Manicheistic idea that good and evil are two equally powerful forces in the world, but that it continued to exercise influence upon his thought—for instance, when it comes to his assessment of the extent to which we can enjoy things in themselves (*frui*) or merely for the sake of some higher good, namely God (*uti*).^42^ Various independent researchers have argued this, in doing so they came up with new data (new passages or new historical background knowledge), they used the same hermeneutical or historical-critical method, and explained the consonance with the original results (and thus the successful replication, even though they would not have used that word) by sketching a larger picture of Augustine’s thought that made sense of his relation to Gnosticism.

Here is another example of a study that employs the hermeneutical method. The crucial difference with the previous example is that this is still a hotly debated issue and that it is not clear exactly what counts as a replication, since it is not clear that advocates and opponents share enough background beliefs in order to properly execute a replication study; only the future will tell us whether that is indeed the case. What I have in mind is the so-called *New Perspective on Paul* in New Testament theology. Since the 1960s, Protestant scholars started to interpret the New Testament letters of Paul differently from how they had been understood by Protestants so far. Historically, Lutherans and Reformed theologians had understood Paul as arguing that the good works of faith do not factor into their salvation—only faith itself would (in a slogan: *sola fide*). The New Perspective, advocated by Ed Parish Sanders and Tom Wright,^43^ however, has it that Paul was not so much addressing good works in general, but specific Jewish laws regarding circumcision, dietary laws, Sabbath laws, and other laws the observance of which set Jews apart from other nations. The New Perspective has been embraced by most Roman Catholic and Orthodox theologians and a substantial number of Protestants theologians, but is still very much under debate. Thus, we should not conclude from the fact that *some* studies that employ the hermeneutic method are replicable that *all* of them are: some of them may involve too many controversial background assumptions in order for a fairly straightforward replication to be possible.

However, it is easy to add examples of studies from other humanistic fields that meet the criterion of replicability. Here are two of them that use a different method than the hermeneutical one:

- The granodiorite stele that was named the Rosetta Stone and that was found in 1799, has texts both in Ancient Egyptian, using hieroglyphic and Demotic script, and an Ancient Greek text. The differences in the content of these three texts are minor. The stone has turned out be the key in deciphering Egyptian hieroglyphs. A large number of scholars have studied the stone in detail and the most important results have been replicated multiple times.^44^
- It was established in 2013 by way of various methods—such as study of the materials, chemical composition, painting style, and a study of his letters—that the painting *Sunset at Montmajour* is a true Van Gogh. It was painted on July 4, 1888. If one has the right background knowledge and skills, one can fairly easily study the same data or collect further data in order to replicate this study.^45^

I take the examples given so far to be representative and, therefore, to provide an inductive argument for the possibility of replication in the humanities: it turns out that in a variety of humanistic fields that employ different methods replication is possible.

Now, the KNAW Advisory Report *Replication Studies* mentions three things to pay attention to in carrying out a replication study: (i) look at the raw data, the final outcomes (results/conclusions) and/or everything in between, (ii) take a rigorous statistical approach or a more qualitative approach in making the comparison between the original study and the replication study, and (iii) define how much similarity is required for a successful replication. This is important, for it means that even the specific way in which a replication study is supposed to be carried out can be copied in a replication study in the humanities. After all, it is possible (i) to compare the original data (say, certain texts, archeological findings, the occurrence of certain verbs, and so on), the conclusions of the original study and the replication study, and everything in between, (ii) to take a qualitative approach and sometimes even, if not a rigorous statistical approach, at least a more quantitative approach, e.g., by counting the number of verbs in Shakespeare’s plays that end in “th” or “st,” and (iii) to define how much similarity between the original results and the results in the replication study is required for something’s being a successful replication, even though this will be harder or impossible to *quantify*, in opposition to many studies in, say, psychology and economics.

## The desirability of replicability and replication in the humanities

It is widely agreed that replicability is a desideratum and replication an epistemically positive feature of a study in the quantitative empirical sciences. Given that, as we have seen in the preceding sections, replication is possible in the humanities, is it something we should pursue? Should we desire that studies be replicable and that a significant number of them be replicated, that is, that they are indeed replicated with a positive, confirming outcome?

The answer has to be: Yes. After all, if, as I argued, replication is possible in the humanities and consistent replication makes it likely that the results of the original study are true, then carrying out such replication studies contributes to such core epistemic aims of the academic enterprise as knowledge, insight, and understanding—which all require truth. Of course, one will have to find the right balance between carrying out *new* research—with, possibly or likely, stumbling upon new truths, never found before—and replicating a study and thereby making it likely that the original study results are true. However, there is nothing special about the humanities when it comes to the fact that we need to find the right balance between various intellectual goals: we need to find the right balance in *any* discipline—medicine, psychology, and economics included. This is not to deny that there may be important differences between various fields. Research indicates that as much as 70% of studies in social psychology turn out not to be replicated upon attempting to replicated them.^46^ This gives us both epistemic reason—it decreases the likelihood of truth of the original study—and pragmatic reason—it defeats public trust in science as a source of knowledge—to carry out more replication studies. Thus, *how much* replication is needed depends on the epistemic state a particular discipline is in.

Certainly, it is not at all common to speak of a “replication crisis” in the case of the humanities, in contrast to some of the quantitative empirical sciences. As various philosophers, such as Martha Nussbaum,^47^ have argued, though, there is at least a crisis in the humanities in the sense that they are relatively widely thought of as having a low epistemic status. They are thought to be not nearly as reliable as the sciences and not to provide any robust knowledge. To give just one example, according to American philosopher of science Alex Rosenberg:

When it comes to real understanding, the humanities are nothing we have to take seriously, except as symptoms. But they are everything we need to take seriously when it comes to entertainment, enjoyment, and psychological satisfaction. Just don’t treat them as knowledge or wisdom.^48^

Another well-known example is the recent so-called grievance studies affair (or hoax). This was an attempt in 2017–2018 by three scholars—James Lindsay, Peter Boghossian, and Helen Pluckrose—to test the editorial and peer review process of various fields in the humanities. They did so by trying to get bogus papers published in influential academic journals in fields such as feminism studies, gender studies, race studies, and sexuality studies. They managed to publish a significant number of papers (which were all retracted after the hoax was revealed), and got an even larger number accepted (without yet being published). However, it is rather controversial exactly what this hoax shows about the epistemic status of these fields in the humanities.^49^ Some have argued that the results would have been similar in pretty much any other empirical discipline,^50^ and still others that we cannot conclude anything from this hoax, since there was no control group.^51^

In any case, there may well be a crisis in how the humanities are perceived. Yet, there does not seem to be a *replication crisis*—at least, it is usually not framed as such. There may, therefore, be somewhat less of a social and epistemic urge to carry out replication studies in the humanities. However, given the epistemic and pragmatic reasons to do so, carrying out at least *some* replication studies would be good for the humanities and for how they are publicly perceived.

We should also realize that one of the reasons that people started to talk about a replication crisis in certain empirical sciences in the first place was that, apart from problems with replicability (some studies did not even meet that desideratum), for some studies an attempt at replication took place but was unsuccessful, so they met replicability as a desideratum, but *not* the positive property of replication. That showed the *need* for more replication studies. Thus, one way to discover the need for replication studies is, paradoxically, to carry out such replication studies. This means that, in order to establish the extent to which replication studies are needed in various fields in the humanities, we should simply carry them out.

Before we move on, I would like to discuss an objection against the desirability of replication in the humanities. The objection is that even though replication may well be *possible* in the humanities, it is not particularly desirable—not something to aim at or invest research money on—because there is simply too much disagreement in the humanities for there to be a successful replication sufficiently often. Thus, even though many humanistic studies would be replicable, carrying out a replication study would in the majority of cases lead to different results. In philosophy, for instance, there is a rather radical divide between scholars in the analytic tradition and scholars in the continental tradition. One might think it likely that a replication of any study by members of the one group would lead to substantially different results if carried out by members of the other group.

We should not forget, though, that we find radically different sorts of schools within, say, economics or physics. In economics, for instance, we find the economics of the Saltwater school, the economics of the Freshwater school, and, more rarely, institutional economics, Austrian economics, feminist economics, Marxian economics, and ecological economics. In quantum mechanics, we find a wide variety of different interpretations with different ideas about randomness and determinacy, the nature of measurement, and which elements in quantum mechanics can be considered real: the Standard or Copenhagen interpretation, the consistent histories interpretation, the many worlds interpretation, the transactional interpretation, and so on.

The problem that this objection draws our attention to, then, is a general one: if a study from one school of thought is replicated by members of a different school of thought, it is much more likely that relevant background assumptions will be different and various auxiliary hypotheses will play an important role. This may make it easier for the researchers of the original study to reject the results of the new study if they differ from those of the original one: they may point to different background assumptions and different auxiliary hypotheses. That does not necessarily undermine the value of those replication studies, though revision in background assumptions or change in auxiliary hypotheses may be widely considered to be an improvement in comparison with the original study or a legitimate change for other reasons. Also, even if the study’s background assumptions are different and various auxiliary hypotheses differ, the study may still be successfully replicated.

The most important point to note here, though, is that, to the extent that this is a problem (and we have seen that it is not necessarily a problem at all), it is a *general* problem and not one that is unique to the humanities.

This is not to deny that there may be situations in which there is *too much divergence* on background assumptions, method, relevant auxiliary hypotheses, and so on, to carry out a replication study. This will be the case for *some* humanistic studies and research groups, as it will be the case for *some* scientific studies and research groups. What this means is that in some humanistic disciplines, replicability is still a desideratum and replicability surely is still a positive property, but the *absence* of replicability because of severe limits on the possibility of replication is not necessarily a reason to discard that study. In other words, in balancing the theoretical virtues of various hypotheses and studies in the humanities, replicability will sometimes not be weighed as heavily as, say, consistency with background knowledge, simplicity, internal coherence, and other intellectual virtues. That is, of course, as such not a problem at all, as the weight of various intellectual virtues differs from discipline to discipline anyway; predictive power, for instance, is crucial in much of physics, but carries much less weight in economics and evolutionary biology.

## Conclusions

I conclude that *replication* is *possible* in the humanities. By that, I mean that empirical studies in the humanities are often such that an independent repetition of it, using similar or different methods and conducted under similar circumstances, can be carried out. I also conclude that *replicability* is desirable in the humanities: by that, I mean that many empirical studies in the humanities should indeed be such that an independent repetition of it, using similar or different methods and conducted under similar circumstances, can be carried out. And I conclude that *carrying out replication studies* in the humanities is desirable: we should actually frequently carry out such independent repetitions of published studies. Exactly how desirable replication in the humanities is remains to be seen; paradoxically, carrying out replication studies in the humanities may tell us more about exactly how desirable doing so is.

## Footnotes

1 See Begley [2].

2 See Open Science Collaboration [3].

3 See Baker [4]; Ioannidis [5]; Nuzzo [6]; Munafò and Smith [7].

4 See http://www8.nationalacademies.org/cp/projectview.aspx?key=49906, last visited May 1, 2018.

5 For full bibliographical details, see the list in the “References” section.

6 For overviews of such causes, see AMS [8], 5, 16–21; IAP [9], 1; KNAW, 23–24 [10]; Munafò et al. [11], 2. Bouter [12] further analyzes the causes for various kinds of questionable research practices. For the issue of lack of *effective* peer review, see, for instance, [13]. In this paper, Smith argues that there is actually no systematically acquired evidence for thinking that peer review is a good quality assurance mechanism and that we *do* have good evidence for thinking that peer review has a large number of downsides.

7 For these points, see also KNAW [10], 4, 20–22.

8 Note the KNAW Advisory Report’s subtitle: *Improving Reproducibility in the Empirical Sciences*.

9 KNAW [10], 16.

10 See [14, 15], and a recent co-authored blog: [16]. The paper at hand provides a much more in-depth exploration of the ideas about replication in the humanities advocated in these three pieces.

11 The humanities are to be distinguished from the *sciences*, where I take the sciences to include the *applied* sciences, such as medicine, engineering, computer science, and applied physics, the *formal* sciences, such as decision theory, statistics, systems theory, theoretical computer science, and mathematics, the *natural* sciences, such as physics, chemistry, earth science, ecology, oceanography, geology, meteorology, astronomy, life science, biology, zoology, and botany, and the *social* sciences, such as criminology, economy, and psychology.

12 I would be happy, though, to embrace the definitions given of these terms in the KNAW Advisory Report, viz. for “robustness”: the extent to which the conclusions depend on minor changes in the procedures and assumptions, for “reliability of measurements”: the measurement error due to variation, and for “verifiability of results”: the extent to which the study documentation provides enough information on how results have been attained to assess compliance with relevant standards (cf. [10], 19). As will become clear from what follows in this section, the phenomena of robustness, reliability, and verifiability, thus understood, are in interesting ways *related to*, but nevertheless clearly conceptually *distinct from* replication, replicability, reproduction, and reproducibility.

13 Some people use the word “reproduction” somewhat more narrowly, namely merely for a study that re-analyzes the same data of the original study and scrutinizes whether they lead to the same results. I will use a broader definition here.

14 For a similar definition, see KNAW [10], 18; NSF [17], 4–5. IAP [9], unfortunately, provides no definition.

15 A fourth option, not mentioned in the report, is to carry out a replication with *the same data* and *a new or revised research protocol*.

16 For the purposes of the paper, I take a “research protocol” to be primarily a description of the study design: a description of which data are taken to be relevant and which method is used.

17 Italics are mine. See https://www.nwo.nl/en/funding/our-funding-instruments/sgw/replication-studies/replication-studies.html, last visited August 30, 2018. For the different kinds of replication, see also [18].

18 The KNAW [10] report, for instance, does not.

19 See LeBel et al. [19], 9; see also Earp and Trafimow [20].

20 Thus, for instance, KNAW [10], 18: “reproducibility concerns the extent to which the results of a replication study agree with those of the earlier study.”

21 For example, LeBel et al. [19].

22 See, for instance, Popper [21].

23 See [22]. As to the role of auxiliary assumptions, such as ones about the role of language, he also gives a particular example that illustrates this claim—one about walking speed in response to being primed with the elderly stereotype (the original study being [23]). For further illustrations of the fact that direct falsification of a theory is virtually impossible, see [20, 24].

24 See Earp and Trafimov [20]

25 This is not to deny that Popper himself thought falsification to be a good thing, since he believed scientific progress to consist of instances of falsification (see [25], 215–250).

26 For example, AMS [8], 9.

27 This approach to agreement on results squares well with that of [26], and that of [27]. I thank Lex Bouter for helpful suggestions on this point.

28 KNAW [10], 33

29 This is also noted in the KNAW [10] Report, 18, 25, and spelled out in more detail in [19], 14. For more on the nature of degrees, that is, for what I take it to be for something to come in degrees, see [28].

30 See LeBel et al. [19]

31 Thus, also KNAW [10], 4, 19.

32 This worry is also found in the KNAW Advisory Report: KNAW [10], 17, 29, even though it is pointed out that this worry might not be decisive. We find the same idea among certain neo-Kantians; see, for instance, [29].

33 See, for instance, Payne [30].

34 Moreover, one may wonder whether there are such things as *unique* historical events studied by historians. One might think, for instance, that the French revolution is not a unique historical event, but just a series of (virtually) infinitely many smaller events, and that history always studies a combination of those events rather than a single, unique event.

35 This was concluded by a recent study; see [31].

36 For example, Lorenz [32].

37 In a way, then, replication—including replication in the humanities—is like what mathematicians do in checking a proof and lay people in checking a particular calculation (say, splitting the bill in a restaurant); if a large number of competent people come to the same result, then, all else being equal, the result is likely to be true.

38 See [33], 112–122. This is not to deny that they may have meaning or significance in *some* sense; the double-helix structure of DNA may be of special significance to, say, James Watson, Francis Crick, and Rosalind Franklin.

39 For a defense of this position, see [34].

40 For an exploration and discussion, see, for instance, [35].

41 For a more detailed exposition and discussion of the hermeneutical method, see [36, 37].

42 For this interpretation, see various essays in Van [38]; and [39].

43 See Sander [40] and Wright [41].

44 For an overview of much research on the Rosetta stone, see [42].

45 See Van Tilborgh, Meedendorp, Van Maanen [43].

46 See, for instance, Klein [44].

47 See Nussbaum [45], chapter 1.

48 Rosenberg [34], 307.

49 See Lindsay et al. [46]. For a more positive assessment, see Mounk 2018 [47].

50 See Engber 2018 [48].

51 See [49]. For a defense of the hoax on these two points, see Essig, Moorti 2018 [50].
